# HIV-1 Tat Binding to PCAF Bromodomain: Structural Determinants from Computational Methods

**DOI:** 10.3390/biology1020277

**Published:** 2012-08-13

**Authors:** Vo Cam Quy, Sergio Pantano, Giulia Rossetti, Mauro Giacca, Paolo Carloni

**Affiliations:** 1 Computational Biophysics, German Research School for Simulation Sciences, Computational Biomedicine, Institute for Advanced Simulation (IAS-5), Forschungszentrum Jülich, Jülich D-52425, Germany; Email: v.c.quy@grs-sim.de (F.L.); g.rossetti@grs-sim.de (G.R.); 2 Institut Pasteur de Montevideo, Mataojo 2020, Montevideo CP 11400, Uruguay; Email: spantano@pasteur.edu.uy; 3 Institute for Research in Biomedicine and Barcelona Supercomputing Center Joint Research Program on Computational Biology, Baldiri I Reixac 10, Barcelona S-08028, Spain; 4 Jülich Supercomputing Centre, Forschungszentrum Jülich, Jülich D-52425, Germany; 5 International Centre for Genetic Engineering and Biotechnology, Trieste 34149, Italy; Email: giacca@icgeb.org

**Keywords:** Tat, PCAF BRD, HIV-1, docking, protein-protein interaction

## Abstract

The binding between the HIV-1 *trans*-activator of transcription (Tat) and p300/(CREB-binding protein)-associated factor (PCAF) bromodomain is a crucial step in the HIV-1 life cycle. However, the structure of the full length acetylated Tat bound to PCAF has not been yet determined experimentally. Acetylation of Tat residues can play a critical role in enhancing HIV-1 transcriptional activation. Here, we have combined a fully flexible protein-protein docking approach with molecular dynamics simulations to predict the structural determinants of the complex for the common HIV-1_BRU_ variant. This model reproduces all the crucial contacts between the Tat peptide ^46^SYGR(AcK)KRRQRC^56^ and the PCAF bromodomain previously reported by NMR spectroscopy. Additionally, inclusion of the entire Tat protein results in additional contact points at the protein-protein interface. The model is consistent with the available experimental data reported and adds novel information to our previous structural predictions of the PCAF bromodomain in complex with the rare HIV_Z2_ variant, which was obtained with a less accurate computational method. This improved characterization of Tat.PCAF bromodomain binding may help in defining the structural determinants of other protein interactions involving lysine acetylation.

## 1. Introduction

**Figure 1:**
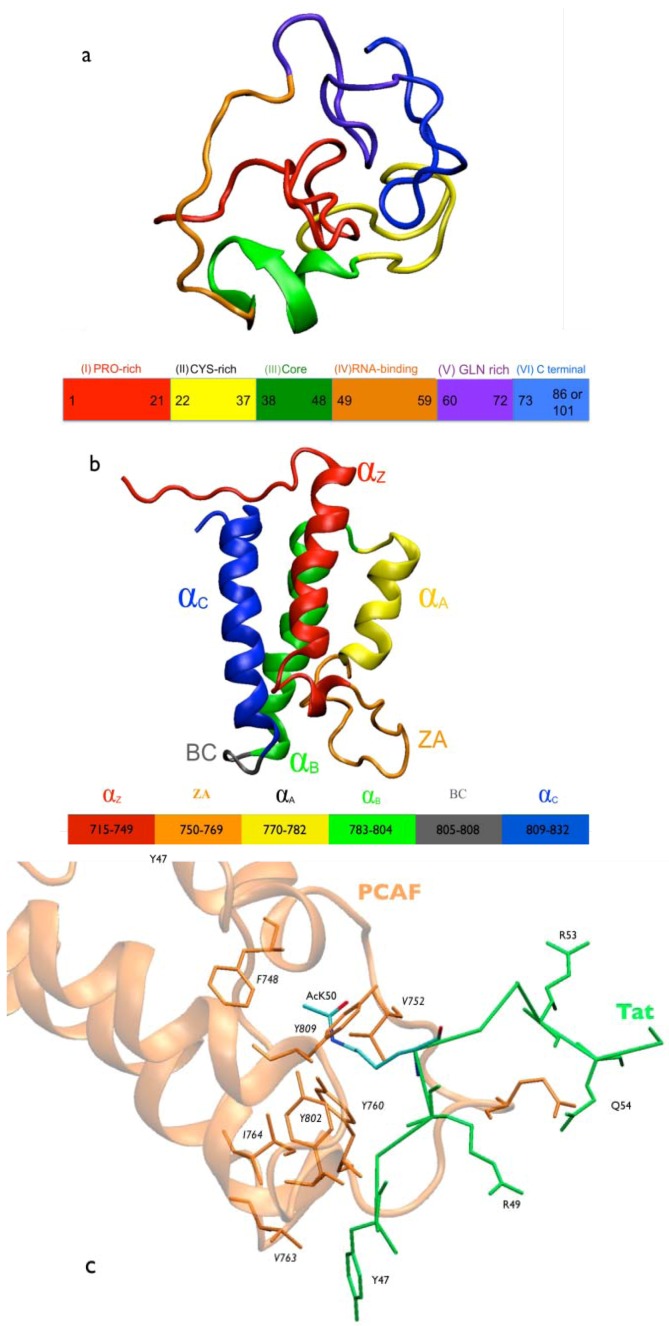
Molecular representation of Tat.PCAF model and interactions. (**a**) HIV-1 Tat protein. The Tat protein lacks secondary structure elements [13]. Tat consists of six regions: Region I (in red) includes a proline-rich tract. It plays an important role in the entry of Tat into the cell [36]. Region II (in yellow) includes a cystein-rich tract. It is important for the transactivation ability of Tat [37]. Region III (in green) includes the motif RKGLGI. It is considered as the hydrophobic core of the protein and is conserved across HIV-1, HIV-2, and SIV Tat [38]. It does not have any known specific function [38]. Region IV (in orange) contains the basic domain (^49^RKKRRQRR^56^), arginine-rich motif, conserved across Tat proteins [39,40]. This region is required for binding to the viral RNA TAR (trans-activating response region) [41,42,43,44,45], as well as a variety of proteins, including PCAF BRD [18]. Region V (in violet) is a glutamine-rich region [46]. It is involved in microtubule polymerization and Tat-mediated apoptosis of T-cells [46]. Region VI (in blue, amino acid 73 to 86 or 101, depending on the virus strain) [10,11] includes the RGD motif that mediates Tat binding to cell surface integrins [47]. It is involved in cell adhesion [48]; (**b**) PCAF BRD. Cartoon representation of PCAF BRD NMR structure (PDBID 1JM4 [18]). It has a four-helix bundle (helices αZ, αA, αB, and αC); (**c**) Tat’s core domain and arginine-rich motif (ARM) interacting with PCAF BRD NMR structure [18]. PCAF BRD (orange) is represented in cartoon and traced with the residues in contact with Tat (green) in orange stick forms. Tat is represented in stick forms with oxygen and nitrogen atoms of AcK50 in red and blue, respectively.

The development of drugs against human immunodeficiency virus type 1 (HIV-1) was initiated about 30 years ago [1]. Since then, there have been 30 available antiretroviral drugs approved by the U.S. Food and Drug Administration (FDA). Current therapies against the AIDS pandemic are mainly based on inhibition of the three enzymes expressed by HIV-1 (protease, reverse transcriptase and integrase) [2,3,4,5]. Unfortunately, all of these approaches suffer from drug resistance due to the emergence of viral mutants [6]. In a rapid process of Darwinian evolution, the virus has developed functional enzyme variants not inhibited by current therapies/drugs. Several promising new strategies may, at least in part, counteract such viral hypervariability [7,8]. One of these strategies interferes with the HIV-1 transcriptional activity induced by the HIV-1 *trans*-activator of transcription (Tat) protein [9,10,11] (Figure 1a). Tat is a small and flexible protein, which acts as a molecular adaptor. Nuclear magnetic resonance (NMR) experiments reveal no secondary structure elements for free Tat in solution [12,13,14,15,16], although dramatic structural changes have been observed (both X-ray and NMR structures) when the protein is in complex with host cell partners [17,18]. The high plasticity of Tat is demonstrated by the fact that it can tolerate up to a 40% of sequence variation without loss of activity [19]. 

Post-translational modifications—in particular acetylation of Tat residues can play a critical role in HIV replication, enhancing HIV-1 transcriptional activation [20,21,22]. 

Tat is acetylated at K50 by the p300/CPB co-activator and histone acetyltransferase (HAT) [10,11,21,23,24,25]. The p300/CPB protein contains several functional modules, including a nuclear receptor interaction domain (RID), a CREB and MYB interaction domain (KIX), cysteine/histidine regions (TAZ1/CH1 and TAZ2/CH3), an interferon response binding domain (IBiD), a histone acetyltransferase (PAT/HAT) domain [20,26,27,28,29], and a motif called the bromodomain (BRD). BRDs have recently been discovered to function as acetyl-lysine binding domains [30]. They feature a characteristic left-handed, four-helix bundle (helices αZ, αA, αB, and αC (Figure 1b). A large loop links helices αZ and αA (ZA loop), and a shorter loop links helices αB and αC (BC loop) [23]. Acetylated lysines bind between both loops by inserting the acetyl moiety along the longitudinal axis of the helices and between the four helices bundle [23,25,30]. In all the structures of BRDs in complex with cognate histone peptides so far determined [31], the acetyl moiety forms a hydrogen bond with a very conserved asparagine residue at the end of the cavity, serving as an anchor and providing specificity for acetylated *versus* non acetylated lysines [32]. Additional contacts with flanking amino acids in the target motif determine specificity/affinity for the large variety of BRD modules so far reported [31]. It is worth noticing that the binding regions belong to post-transcriptional modified histone tails, which are intrinsically disordered [33,34,35]. Therefore, it is not clear whether additional contacts may contribute to the recognition process. 

Tat is also acetylated at K28 by the p300/CBP-associated factor (PCAF), thereby enhancing Tat binding to the protein P-TEFb [17]. This may in turn regulate functionally critical steps in transcription [20,21,22,49,50,51]. PCAF is a 90-kDa protein that contains the P300/SRC-1 domain (residues 1 to 352), the HAT domain (residues 518 to 649), which acetylates K28 [18,51] and a C-terminal BRD (residues 719-832) [18]. The BRD binds to the Tat protein, which is acetylated at lysine 50 (AcK50). Despite the fact that the structural determinants of full-length PCAF in complex with Tat have not yet been determined experimentally, the NMR structure of the complex between an acetylated Tat peptide (^46^SYGR(AcK)KRRQRC^56^) (Figure 1c) and the PCAF BRD has been solved by NMR spectroscopy [18].

The structural determinants of the full-length Tat PCAF BRD complex were predicted based on molecular dynamics simulations, combined with biochemical and FRET experiments [52]. This model was built using the structure of the isolated Z2 variant of the HIV-1 Tat [15]. In contrast to the existing structures of BRDs in complex with histone peptides, that model pointed to the presence of contacts between the BRD and non-adjacent regions of the acetylated partner. Furthermore, Tat Regions III and IV made extensive contacts with the ZA loop of the PCAF BRD (Figure 1). Although these contacts were not present in the NMR structure, which only contained a Tat peptide, they were experimentally validated [52], supporting the idea that significant changes in the protein-protein interface may take place in the presence of the entire Tat molecule. 

Here we construct an updated structural model of the Tat PCAF BRD complex using a more accurate docking procedure. This is the fully flexible protein-protein technique as implemented in the data-driven docking program HADDOCK version 2.1 [53,54] followed by molecular dynamics (MD) simulations. Importantly, we focused on the 86 amino acids Tat BRU variant, PDBID 1JFW [13], a representative of HIV-1 subtype B. This variant is actually the more common HIV subtype in Europe and North America [12]. The Z2 variant of the protein used in our previous study is evolutionally closer to early strains of the virus, but is nowadays much less abundant [12]. Docking studies involving Tat and its cellular partners can be expected to be feasible. Indeed, (i) HADDOCK (High Ambiguity Driven protein-protein DOCKing) [53,54] has been used to predict the protein-protein and protein-nucleic acid complexes based on available experimental data [55,56,57]; (ii) HADDOCK has also been applied to intrinsically disordered proteins for which NMR structural information is available, such as Tat [50,51,52,53]; (iii) HADDOCK has been successfully applied to a variety of NMR structures with a low number of NOE’s per atom [54,55], similar to the ones we found in the available Tat NMR structures. Moreover, HADDOCK complemented by MD also provides reasonable results in those cases where the protein undergoes conformational changes [56,57,58]. Therefore by using the docking procedure combined with the MD simulations, we believe that a reasonable model of Tat can be assured.

## 2. Results and Discussion

### 2.1. Intermolecular Contacts

In our full-length Tat.PCAF BRD model, the interface between the BRU Tat and PCAF BRD complex (BRU Tat.PCAF hereafter) consists of Tat residues 47–55 and PCAF BRD residues 722–781. We focus on the contacts at the protein/protein interface. We first describe the hydrophobic contacts (HCs). Then we move our attention to intermolecular hydrogen bonds (HBs) and salt-bridges (SBs). The residues belonging to PCAF BRD are indicated in the following text with italics, while those of Tat with normal text.

**Figure 2:**
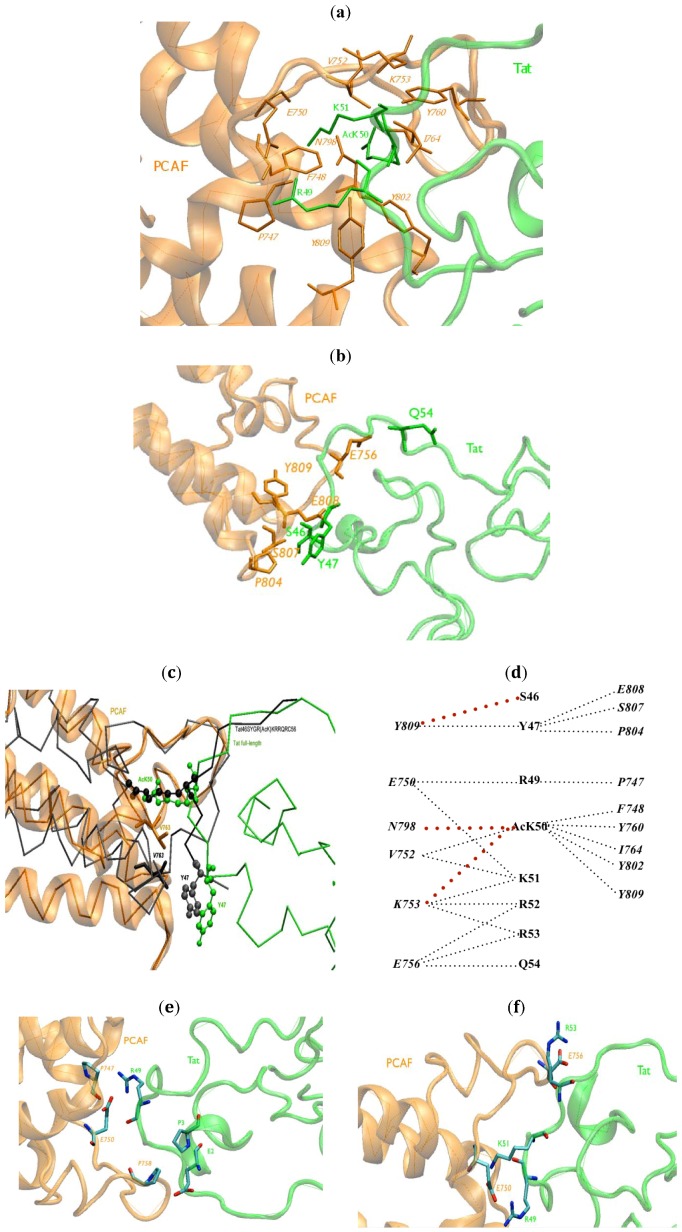
BRU Tat.PCAF BRD interface (**a**) HCs between R49, AcK50, R51 of BRU Tat (green) and PCAF BRD (orange) contacts at the core protein/protein interface are reported. Residues at the interaction interface are represented in stick forms; (**b**) The HCs between BRU Tat (green) and PCAF BRD (orange) contacts at the edge protein/protein interface are reported. Residues at the interaction interface are represented in stick forms; (**c**) The position of Y47 (shown with balls) and *V763* (shown with sticks) represented in the complex of BRU Tat.PCAF model were superimposed with the same residues in Tat^46^SYGR(AcK)KRRQRC^56^.PCAF (black) NMR structure [18] (in green and orange, respectively); (**d**) A schematic view of the HCs between BRU Tat and PCAF BRD. The red dotted lines are the HCs newly found in BRU Tat.PCAF model; (**e**) The new HBs in BRU Tat.PCAF model (in green and orange, respectively) with oxygen and nitrogen atoms in red and blue, respectively; (**f**) salt-bridges (SBs) between BRU Tat (green) and PCAF (orange) between R53–*E756*, R49–*E750*, K51–*E750.*

AcK50 forms HCs with *F748*, *V752*, *K753*, *Y760*, *I764*, *N798*, *Y802* and *Y809* (Table 1 and Figure 2a). These results are in agreement with *in vitro* mutagenesis experiments in which the mutants to alanine of these PCAF residues strongly dismissed the Tat.PCAF BRD binding [18]. The role of *Y760* in binding has been confirmed also *in vivo* by fluorescence resonance energy transfer (FRET) experiments of full-length acetylated Tat with PCAF *BRD* [52]. The mutant *Y760D* indeed impaired Tat binding to PCAF BRD (Table 1) [52]. Q54 establishes HCs with PCAF *E756*. Both residues are located at the edge of a hydrophobic cavity of Tat (Figure 2b). 

R49 forms HCs with *P747*, *E750*; K51 with *E750*, *V752*, *K753*; R52 with *K753*, *E756* (Figure 2a); and Y47 with *P804*, *S807*, *E808* and *Y809* (Figure 2b). These results are also consistent with *in vitro* mutagenesis experiments [18]; alanine substitution of residues R49, K51, R52, or R53 slightly weaken Tat’s binding to the BRD [18]. Similarly, the mutation of Y47 and Q54 to alanine hampers PCAF BRD binding to the Tat^46^SYGR(AcK)KRRQRC^56^ peptide. Most of these HCs are present in the Tat^46^SYGR(AcK)KRRQRC^56^.PCAF NMR structure [18] (Table 1). Additionally, our model predicts interactions between AcK50–*K753*, AcK50–*N798* (Figure 2a) and S46–*Y809* (Figure 2b); the last predicted interaction is possibly because residues flanking both sides of S46 contribute to the complex formation (Figure 2b). In addition, the *V763*–Y47 contact is present in the NMR structure only and absent in our model (Figure 2c). This is due to the fact that the formations of new HCs at the N-terminus affect the position of Y47 in the BRU Tat.PCAF model. The new HCs include Tat E2 to PCAF *E756*, *A757*, *P758*; P3 to *P758*; S46 to *P804*, *Y809*. As a result, the RMSD of Tat Y47 exhibits a difference of 4.8 Å with respect to the full length Tat.

**Table 1:** Effect of mutations in *in vitro* experiment for Tat^46^SYGR(AcK)KRRQRC^56^ (synthesis) complex with p300/(CREB-binding protein)-associated factor (PCAF) bromodomain (BRD) [18] or *in vivo* experiment for full-length acetylated Z2 Tat with PCAF BRD [52]. The corresponding contacts in both BRU Tat.PCAF docking and molecular dynamics (MD) models presented in this work are reported in the last columns (black = agreement; red = new contacts). Coverage in the last column is the percentage of the occurrence of hydrophobic contacts (HCs) over all frames in MD simulation.

Effect on Tat.PCAF binding	Mutants	In contact with	BRU Tat.PCAF model (docking)	BRU Tat.PCAF model (MD)	Coverage (%)
inhibiting binding [18]	AcK50A	*F748*, *V752*, *Y760*, *I764*	*F748*, *V752*, *Y760*, *I764*	*F748*, *V752*, *Y760*	100%
		*Y802*	*Y802*	*Y802*	80%
		*Y809*	*Y809*	Absent	
			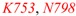	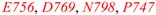	100%
inhibiting binding [18]	Y47A	*V763*	Absent	Absent	
	*V763*	Y47	*P804*, *S807*	*N803*	80%
			*E808*	*E808*	100%
				*Y809*	80%
	Q54A	*E756*	*E756*	*E756*	80%
	*E756A*	Q54	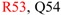	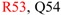	80%
strongly diminishing binding [18]	R53E	*E756*	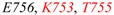	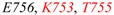	100%
	*F748A*	Tat^46^SYGR(AcK)KRRQRC^56^	AcK50	AcK50	100%
	*V752A*	Tat^46^SYGR(AcK)KRRQRC^56^	AcK50, R51	AcK50, R51	100%
	*Y802A*	Tat^46^SYGR(AcK)KRRQRC^56^	AcK50	AcK50	100%
	*Y809A*	Tat^46^SYGR(AcK)KRRQRC^56^	AcK50, S46	S46	100%
Diminishing binding [18]	R49A	PCAF BRD	*P747*, *E750*	*W746*, *P747*, *E750*, *Y802*	100%
				*F748*	80%
	K51A	PCAF BRD	*E750*, *V752*, *K753*	*F748*, *E750*, *V752*, *E756*	100%
				*K753*	50%
	R52A	PCAF BRD	*K753*	*K753*	25%
			*E756*	*E756*	100%
Diminishing binding [52]	*Y760D*	AcK50	AcK50	AcK50	100%
	*Y761D*	AcK50	AcK50	Absent	
no effect [18]	*W746A*, *D769A*, *C799A*, *N803A*			R49	100%
	*E750A*		R49	R49	100%
	*T755A*		R53	R53	100%
	*I764A*		AcK50	AcK50	100%
	*N798A*		AcK50	AcK50	100%

A schematic view of the HCs between Tat and PCAF BRD is shown in Figure 2d.

Tat AcK50 forms HBs with *V752*, *F748*, *N798*, *Y802* and *Y809*. Tat R53 and Tat Q54 make HBs with PCAF *E756* (Figure 2e) (Table 2). These HBs are also present in the Tat^46^SYGR(AcK)KRRQRC^56^.PCAF NMR structure [18] (Figure 2e) and are consistent with our model of Z2 Tat.PCAF [52]. 

New HBs between R49 and *E750*, *P747* were found in our model (Table 2) at the interface. In the N-terminal of Tat, E2 and P3 make a HB to PCAF *P758.*

Finally, the R53–*E756*, R49–*E750*, K51–*E750* SBs are present (Figure 2f) in the BRU Tat.PCAF BRD model. These residues are all at the interface of Tat and PCAF with the high density of basic/acidic residues. Only the first SB was present in Tat^46^SYGR(AcK)KRRQRC^56^.PCAF NMR structure [18]. This SB was also present in our previous model [52]. 

The five representative clusters of Tat.PCAF complex from MD simulation are compared with the docked model and the NMR structure. The HCs are shown in Table 1. The structures obtained by docking and those obtained by MD are not too dissimilar (Table 1 and Table 2). In particular, some hydrophobic contacts (AcK50–*E756*, AcK50–*D769*, Ack50–*P747*, R49–*W746*, R49–*F748*, R49–*Y802*, K51–*K753*, K51–*E756*) are present in the MD structures but absent in the docked structures. None of the intermolecular HBs with a coverage of more than 50% over the frames is retained in MD. The models generated by data-driven docking and MD show that the hydrophobic interactions may play a leading role in Tat.PCAF BDB complex stabilization. 

**Table 2:** Selected intermolecular hydrogen bonds (HBs) in the BRU Tat.PCAF model by docking.

Donors	Acceptors
AcK50	*V752*
AcK50	*F748*
AcK50	*Y809*
AcK50	*Y802*
*N798*	AcK50
R49 *	*P747* *
R49 *	*E750* *
R53	*E756*
R53	*K753*
R55	*E756*
Q54	*E756*
E2 *	*P758* *
P3 *	*P758* *

* The residues form new HBs in the BRU Tat.PCAF model.

### 2.2. Structural Rearrangements of Tat and PCAF upon Binding

In the Tat^46^SYGR(AcK)KRRQRC^56^.PCAF BRD complex [18], the binding interface between Tat and PCAF BRD is composed of Tat residues 47–55 and PCAF BRD residues 722–781. The inclusion of the complete Tat protein in our model imposes structural constraints that result in conformational differences, which, however, do not significantly alter the identity of the contacts. 

The structural changes mostly involve the Tat BRU variant: The RMSD of the backbone atoms between the Tat^47^YGR(AcK)KRRQR^55^ of our model and the NMR structure of Tat^46^SYGR(AcK)KRRQRC^56^.PCAF BRD [18] is as large as 4 Å. The RMSD of the ^47^YGRKKRRQR^55^segment relative to the Tat^46^SYGR(AcK)KRRQRC^56^.PCAF BRD NMR complex [18] ranges from 2.6 Å to 3.4 Å. A similar result is also found after MD simulations. Indeed, the binding regions (Tat^47−55^ and PCAF^722−781^ BRD) rearrange 3.1 Å and 2.6 Å, respectively, compared to the same residues on NMR structures, while the whole complex shows remarkable changes (RMSD 24.5 Å). This indicates that the binding region rearranges less than the rest of the protein. 

Dramatic changes were instead observed in the N-terminal segment on Tat upon binding with the P-TEFb complex (see Supplementary Section S5 for more details [17]). 

Notably, the RMSD between the present model (using the BRU variant) with that previously reported by us for the Z2 variant [15] (which has 79.1% sequence identity with BRU Tat, see Supplementary Figure S1) is as much as 9.5 Å. The main differences involve the N-termini, with a RMSD of 17 Å dissimilarity between the two structures, and the C-termini which differ by as much as 14 Å; the Tat^46^SYGR(AcK)KRRQRC^56^ is more conserved, with 3.3 Å dissimilarity. The AcK50 in BRU and Z2 Tat.PCAF BRD model was outside the Tat loop such as K50 in Tat full-length BRU variant in unbound conformers. In Z2 Tat full-length, the K50 instead turns inside the loop (See Supplementary Figure S1b). We further notice that in spite of their differences in sequence and overall structure (See Supplementary Figure S1b), the N-termini of Z2 and BRU Tat form similar contacts with PCAF BRD and residues in the N-terminal extremity of Tat. Different segments for the Z2 and BRU variants of Tat are conserved in terms of the intermolecular contacts with PCAF [12], as described in Section 2.1.

The PCAF BRD structure remains close to the protein in the free state in solution: The RMSD of the backbone relative to that of the NMR structure [18] is only 0.8 Å (Table 1).

We further notice that docking of the Tat^46^SYGR(AcK)KRRQRC^56^ peptide onto the PCAF BRD (see Methods Section and Supplementary Section S3) resulted in structural deviations differing by 0.9 Å and 1.3 Å for each of the components and 1.7 Å for the whole complex (Table 3). These values are well within the range of variation of RMSD reported for the NMR structure described in Table 3. This may suggest that our docking procedure is rather accurate. 

**Table 3:** Calculated backbone RMSD values between the BRU Tat.PCAF docking and MD models and the Tat^47^YGR(AcK)KRRQR^55^.PCAF NMR structure (conformer 8 [18], see Methods for details). The abbreviations min., max., avg., sd. stand for minimum, maximum, average, standard deviation of RMSD values, respectively.

Systems	Backbone RMSD (Å):PCAF *722–781*			Backbone RMSD (Å): Tat^47^YGR(AcK)KRRQR^55^			Backbone RMSD (Å): Full complex (same residues)		
	min.	max.	avg.	min.	max.	avg.	min.	max.	avg.
Tat^47^YGR(AcK)KRRQR^55^.PCAF*(1JM4)	0.6	1.5	1.0	0.7	1.2	2.3	0.9	1.9	1.33
	sd. 0.2			sd. 0.5			sd. 0.26		
Tat^47^YGR(AcK)KRRQR^55^.PCAF (Prediction)	1.3			0.9			1.7		
BRU Tat.PCAF (Prediction by docking)	0.8			4.0			14.4		
BRU Tat.PCAF (Prediction by MD simulation, the last frame)	2.9 3.3 3.1 sd. 0.2			2.8 3.4 2.6 sd. 0.4			24.7 25.8 24.5 sd. 1.2		

### 2.3. Key Residues of PCAF BRD for Its Interaction with Tat

The sequence and structure conservation of Tat has been discussed by us in [12]. Here we focus on residues of PCAF human BRD important for Tat binding (See multiple sequence alignment of PCAF human BRD in Supplementary Figure S2). There are 53 PCAF human bromodomain (BRD) sequences as obtained from bromodomain PROSITE family, accession number PS50014 [59,60,61]. We start our discussion by listing the most conserved residues of this protein family (Table 4). In particular, *P758*, which makes HBs with E2 and P3 of Tat, is highly conserved among PCAF BRD sequences (71% identity, Table 4) and *P747* (identity 53% respect to PCAF BRD sequences, Table 4 and Supplementary Figure S2). Notably, the *N798*, which makes an HB with AcK50, is one of the most conserved residues in PCAF BRD (96%, Table 4). *P747* and *N798* residues, which are involved in several HCs and HBs (Table 1 and Table 2 and Supplementary Tables S3 and S4), seem to play an important role for the Tat.PCAF interaction. Moreover, due to the overall structure similarities of PCAF BRD [62], the *N798* may play an important role in the interaction of PCAF BRD with other cellular partners [63]. 

**Table 4:** Conserved residues of the existing 53 PCAF human bromodomain (BRD) sequences as obtained from the bromodomain PROSITE family, accession number PS50014 [59,60,61].

Residues	Identity (%)	Residues	Identity (%)
*P747*	53%	*T772*	69%
*F748*	91%	*Y782*	97%
*P751*	68%	*F788*	63%
*V752*	71%	*D791*	89%
*P758*	71%	*F796*	60%
*I764*	76%	*N798*	96%
*P767*	97%	*Y802*	77%
*D769*	85%	*N803*	88%
*L770*	66%		

## 3. Computational Methods Section

The structure of HIV-1 Tat (86 residues) has been solved by NMR (PDBID 1JFW [13]). Eleven conformers have been deposited. *His13*, *His33*, *His65* are protonated in the Nε [13]. We superimposed residues 47 to 55 in each of these structures with the corresponding ones with the multiple conformers of Tat^46^SYGR(AcK)KRRQRC^56^ peptide in complex with human PCAF BRD, whose structure has been solved by NMR. *His717* and *His742* were protonated in Nδ as in the NMR structure (1JM4 [18]). The VMD 1.8 program [64] was used. The conformer n.8 of HIV-1 Tat and n.3 of the Tat^46^SYGR(AcK)KRRQRC^56^.PCAF complex exhibits the lowest pairwise root-mean-square deviation (RMSD) in respect Tat^46^SYGR(AcK)KRRQRC^56 ^backbone (2.3 Å and 0.7 Å, respectively). All the RMSDs reported in this paper are calculated using only backbone atoms. Thus, these conformers were selected for all subsequent calculations. The full length Tat was fitted to Tat^46^SYGR(AcK)KRRQRC^56^ at the peptide then AcK50 was replaced with K50 in full length Tat using the tleap module in AMBER package [65] with acetylated lysine parameters adapted from Machado *et al.* [66]. The resulting structure was relaxed for 10 ns molecular dynamics simulation in water (See Supplementary Section S1; RMSF, RMSD of the system plotted as a function of simulated time in Supplementary Figure S3 and Figure S4 for further details). A clustering of the MD trajectory [67] identified seven clusters, 84 structures in total, which represented 98% of the conformations (Supplementary Section S2). These structures were docked on the 25 PCAF BRD conformers present in the NMR family of structures (1JM4 [18]) by a multiple conformations docking procedure using the HADDOCK 2.1 program [53,54,68]. Haddock is an information-driven flexible docking approach for the modeling of biomolecular complexes [40]. The entire protocol consists of four stages: (i) Topology and structure generation; (ii) Randomization of the starting orientation and rigid body energy minimization; (iii) Semi-flexible simulated annealing; (iv) Flexible refinement in explicit solvent (water).

We first validated our docking procedure by redocking the Tat^46^SYGR(AcK)KRRQRC^56 ^from PCAF BRD in the NMR structure of the complex (Supplementary Section S3) [18]. Then, the two binding partners were docked one onto the other by rigid body docking with different Ambiguous Interaction Restraints (AIRs) (Supplementary Section S3, Table S2, for the definition of AIRs, see [54]). The adduct exhibited a backbone RMSD of 1.3 Å for PCAF BRD and 1.7 Å for HIV-1 Tat^46^SYGR(AcK)KRRQRC^56^ with respect to the NMR structure (Supplementary Section S3, Figure S4) (Table 3). These results allow us to suggest that HADDOCK performs well for this system (Supplementary Section S3). Next, we performed rigid-body docking between PCAF BRD and Tat AcK50. The contacts *F748*–, *V752*–, *Y809*–, *I764*–, *Y760*–, *Y802*– to AcK50, *E756*– to R53, *V763*– to Y47, *E756*– to Q54, present in the NMR structure [18], were treated as AIRs. The rigid-body docking was followed by a rigid body energy minimization, a semi-flexible simulated annealing in torsion angle space and a refinement by MD at a temperature of 300K in explicit solvent [54,69]. Next we selected the 500 top structures in terms of the highest docking score and the lowest RMSD relative to the NMR structure [18]. In these 500 structures, a short MD simulation was performed to optimize the water positions while proteins were fixed (4,000 steps of MD consisting four times 1,000 steps at a temperature of 600, 500, 400 and 300 K, respectively [69]. This was followed by MD at 300K in water [54]. A clusterization procedure was performed with a cut-off at 3.0 Å from the final 500 structures (94% structures were clusterized). We finally selected the structure that had the lowest RMSD (0.8 Å for PCAF and 4 Å for Tat), which is representative for the most crowded cluster. This is the structure discussed in the Results and Discussion Section. Other representative structures are discussed in Supplementary Table S3.

The representative of the Tat.PCAF model from docking underwent MD simulations in explicit water. Tat.PCAF BRD was solvated in 104 × 70 × 66 Å^3^ box, containing 39.363 water molecules, 16 chlorine ions were added to neutralize the total charge of the system. The AMBER force field ff99SB [70] was used for the protein and the chlorine atoms; the TIP3P model [71] was used for water. The acetylated lysine topology parameter was taken from Machado *et al*. [66]. 

Periodic boundary conditions were used and a cutoff of 12 Å [72] was adopted for short-range non-bonded interactions. The particle mesh Ewald summation method [73] was used for long-range electrostatic interactions. A dielectric constant of one was assumed. All chemical bonds were constrained by using the SHAKE algorithm [74]. The equations of motion were integrated using a time step of 2 fs. Temperature and pressure were kept constant at 300 K and 1 atm by coupling the system to external baths [75] with a Langevin thermostat (coupling constants st = 0.05) and a Berendsen barostat (coupling constants sp = 0.5 ps), respectively.

After 5,000 steps of minimization (steepest descent algorithm for the first 1,500 steps before switching to the conjugate gradient algorithm for the remaining 3,500 steps), the system was equilibrated for 300 ps. Eighty nanoseconds of MD simulation were performed at 300 K and 2 ps for the time steps. We clusterized [67] the trajectory into five clusters. The backbone RMSD of the Tat.PCAF BRD structures over 80 ns is shown in Supplementary Figure S7. NAMD package was used for MD simulation [76].

Hydrophobic contacts at the protein/protein contact surface were defined as two non-polar carbon atoms at a distance of 4.0 Å or less. Hydrogen bonds were defined as a polar hydrogen at a distance of 2.5 Å or less from a polar atom (nitrogen or oxygen). Salt bridges were considered formed if the distance between any of the oxygen atoms of acidic residues and the nitrogen atoms of basic residues were less than 4.0 Å. Coverage was defined as the percentage of the occurrence of HCs over all frames in MD simulation or all representative structures in docking. The HADDOCK [53,54], LIGPLOT [77] and VMD [64] programs were used to calculate these quantities.

## 4. Conclusions

We have presented a model of the BRU Tat.PCAF complex produced by data-driven docking combined with MD simulations, which may add structural information to the intricate mechanism of Tat transcription and trans-activation, up to now not fully understood [10,11]. The structures obtained by docking and those obtained by MD are similar in the binding region. This model was obtained with a more accurate procedure than that used in [52] and it focuses on the more common BRU variant rather than the Z2 strain of HIV-1. The two models show conservation as far as the Tat^46^SYGR(AcK)KRRQRC^56^ and N-terminal contacts with PCAF BRD are concerned. In addition, the model suggests the presence, only for the Z2 strain of Tat, of hydrophobic contacts and hydrogen bonds between Tat and PCAF that are not present in the previous model of Z2 Tat.PCAF [52]. Specifically, the BRU Tat.PCAF model differs from the Z2 Tat.PCAF complex on HBs between R49 and *P747*, *E750*, between *P758* and E2, P3 and on HCs between S46 and *P804*, *Y809*.

The model appears to be consistent with most of the experimental data obtained with Tat^46^SYGR(AcK)KRRQRC^56^.PCAF *in vitro* [18] (synthesized Tat peptide) and full length Tat.PCAF *in vivo* [52]. However, this comparison has to be interpreted with caution because of the assumption that most of the intermolecular interactions compared here do not vary when passing from the *in vitro* to the *in vivo* situation and/or from the Tat^46^SYGR(AcK)KRRQRC^56^ to the full length protein. 

Several BRD amino acids have been identified as crucial for Tat binding (*E750*, *V752*, *E756*, *A757*, *Y802*, *Y809*) and as the targets for development of small molecules blocking Tat.PCAF association [78,79]. Our model allows us to suggest that the hydrophobic interactions seem to be a crucial factor for BRD binding to Tat. 

Further, our model allows us to suggest that *N798* in the BRD plays a key role for Tat binding, independently of whether this protein is expressed by the BRU or the ZZ variant of HIV-1. Indeed, this residue creates a HB with AcK50 in the X-ray structure of Tat and other cellular partners [41,42,49], in both the Z2 Tat.PCAF model [52] and in our model. Hence, interfering with *N798*-AcK50 interactions may be a valuable strategy for drug design [63,80]. Because this residue is highly conserved (Supplementary Figure S3 and Table 4), it may form key interactions also with other BRD cellular partners [18,31,32,62,81]. Moreover, some studies showed that specific small molecules binding at the acetylated lysine recognition pocket can antagonize the bromodomains and acetylate histone interactions, especially the HB between asparagine in αB loop and acetylated lysine [30,80,82,83,84,85,86,87,88,89]. Therefore, designing compounds, which bind to *N798* may also lead to other potential therapeutic applications.

## Supplementary Files

Supplementary File 1 PDF-Document (PDF, 4505 KB)
