# Supplementary material for: HIV-1 Tat Binding to PCAF Bromodomain: Structural Determinants from Computational Methods
Source: Biology (Basel). 2012 Aug 13;1(2):277–96. doi: 10.3390/biology1020277 (PMC4009784; doi:10.3390/biology1020277)
Supplement: Supplementary File 1 — PDF-Document (PDF, 4505 KB) [file biology-01-00277-s001.pdf]

Supplementary Materials

Figure S1. (**a**) Superposition of Tat Z2 variant structure (red, the lowest backbone RMSD on residue 46 to 56 in model 3rd) with Tat BRU strain structure (green, model 8th); the Tat46SYGRKKRRQRC56peptide is in cartoon and K50 is in sticks. (**b**) Sequence alignment and degree of conservation between Z2 Tat and BRU Tat, as obtained by the ClustalW [1] software. Color code as in [1].

a

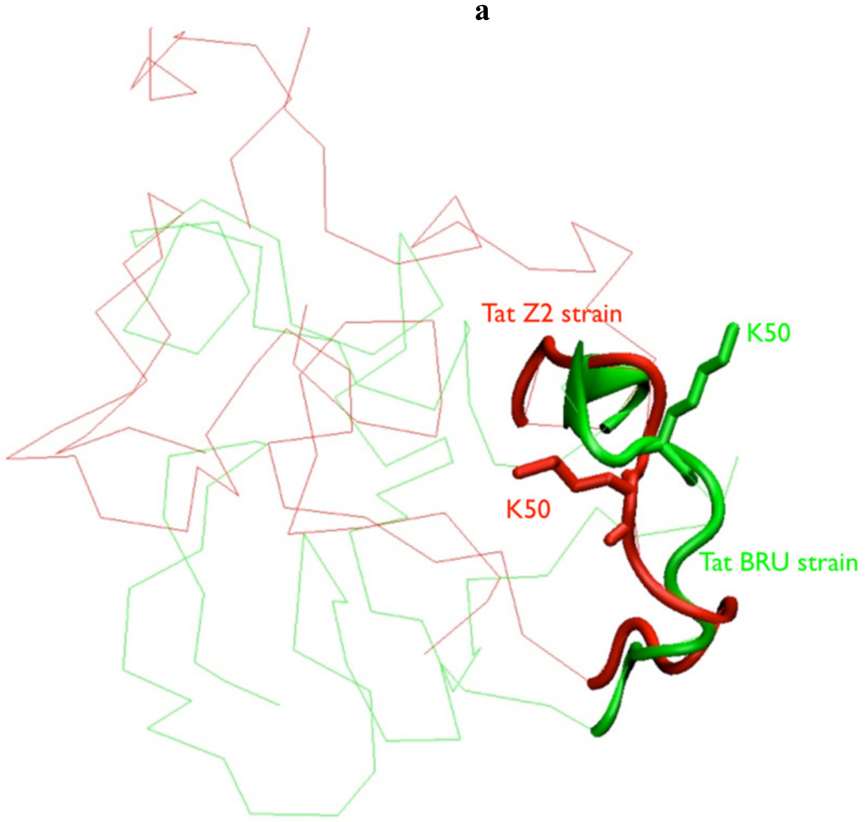

b

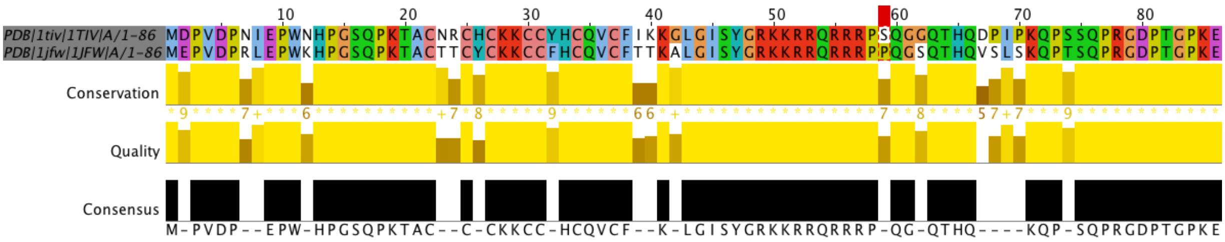

Figure S2. Multiple sequence alignment of the existing 53 PCAF human bromodomain (BRD) sequences as obtained from bromodomain PROSITE family, accession number PS50014 [2–4]. The human PCAF BRD corresponding to the PDB structure 1JM4 is the top sequence. Blue residues in the alignment denote residues that have percentage of identity over 60%. The figure was generated using JalView [5].

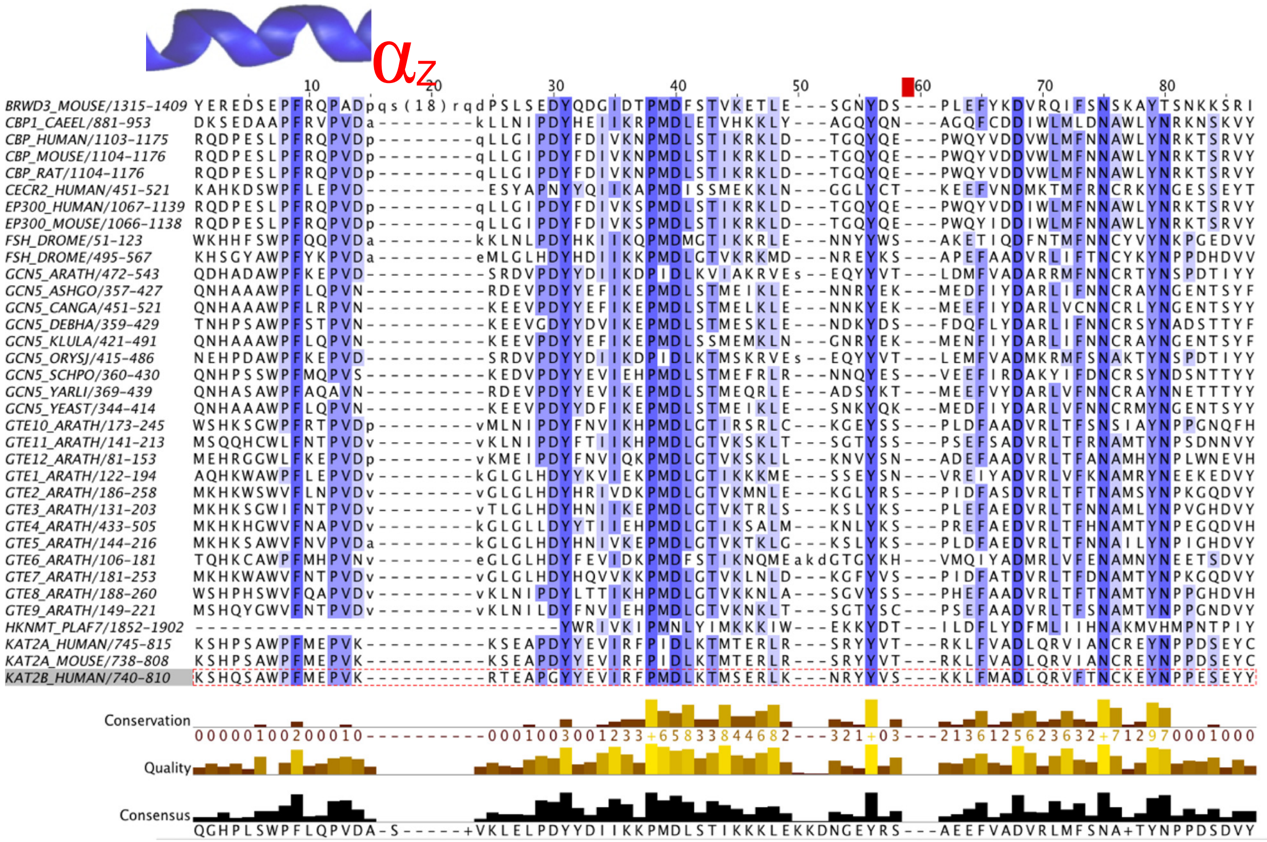

S1. MD Simulation of AcK50-BRU Tat$_{\text{full-length}}$

The of AcK50-BRU Tatfull-length model was inserted in a simulation box of 0.12 nm length containing ~9,300 TIP3P water molecules [6] and 11 Cl^- counterions for neutralizing the system. Periodic boundary conditions were used and a cut-off at 12 Å [7] was adopted for short-range non-bonded interactions. The particle mesh Ewald summation method [8] was used for long-range electrostatic interactions. A dielectric constant of 1 was assumed. All chemical bonds were constrained by using the SHAKE algorithm [9]. The equations of motion were integrated using a time step of 1fs. Temperature and pressure were kept constant at 300 K and 1 atm by coupling the system to external baths [10] with Langevin thermostat (coupling constants st = 0.05) and Berendsen barostat (coupling constants sp = 0.5 ps), respectively. The AMBER force field ff99SB [11] was used. The acetylated lysine topology parameter was adapted according to Machado *et al.* [12].

After 5,000 steps of minimization (steepest descent algorithm for the first 1,500 steps before switching to the conjugate gradient algorithm for the remaining 3,500 steps), 10 ns of MD simulation were performed. NAMD software [13] was used.

We clusterized [14] the trajectory into 7 clusters (See Section S2). For each cluster, 11 representative structures were selected (77 structures in total, grouped in 7 sets) for AcK50-BRU

full-length Tat docking with PCAF BRD. They represent 98% of conformations (Figure S4). Finally, 7 representatives, having at least 3 Å of backbone RMSD from each other, for a total ensemble of 77 structures were chosen.

Figure S3. RMSF of full length AcK50-Tat backbone at 5-6 ns (black); 9-10 ns (red).

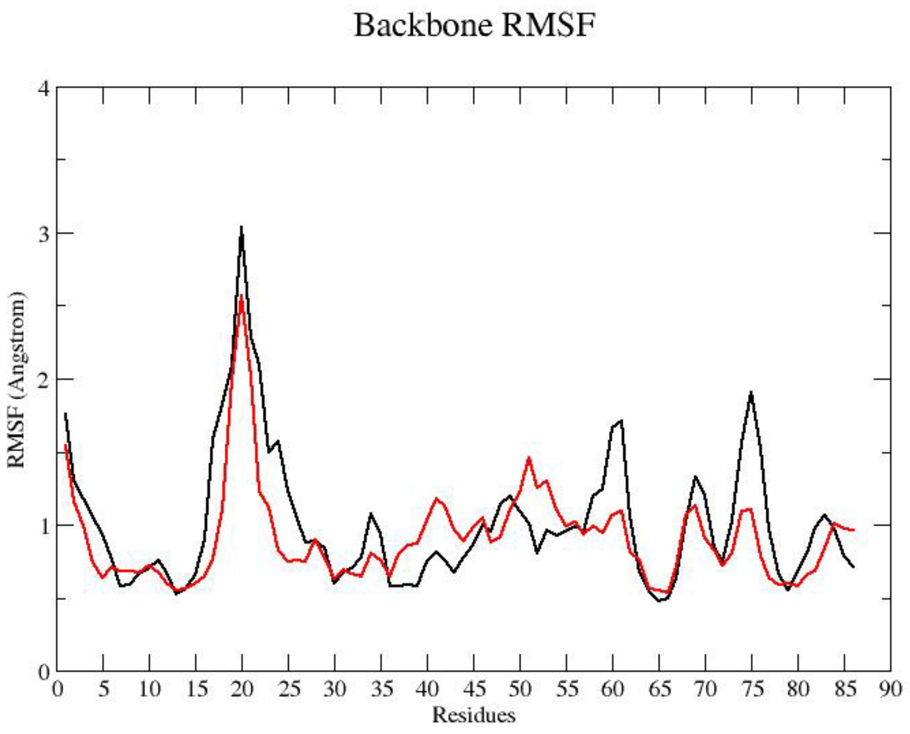

Figure S4. MD simulation of full length AcK50-Tat in water: RMSD of the protein plotted as a function of simulated time.

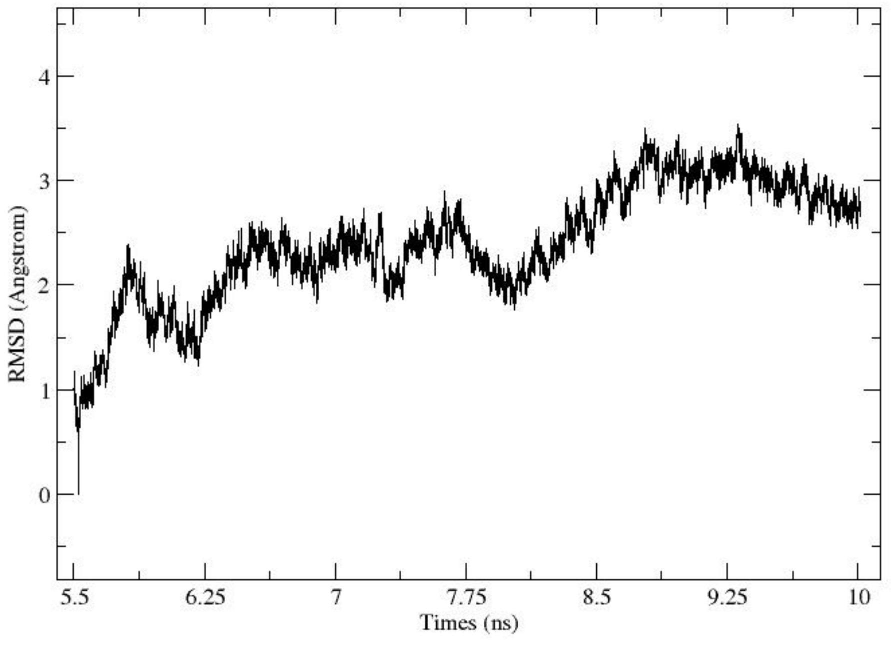

S2. Clusterization Procedure

AcK50-BRU $Tat_{full-length}$ MD conformers were clusterized following the De Mori *et al.* method [45]. We performed the clustering by different cut-offs (Table S1). Cut-off 3.0 Å was chosen as described in Section S1.

Table S1. Performance of different cut-off in clustering MD conformers.

Cut-off (Å)	Number of clusters	Number of structures in the highest clusters	Number of structures in the lowest clusters
2.0	15	1182/875/748/427/257	1/5/6
2.5	9	2035/915/518/313/116	3/6/7/16
3.0	7	2489/1199/188	9/13/31

S3. Validation of Docking Parameters

Different Ambiguous Interaction Restraints were used to find the best parameters for docking (Table S2).

Table S2. Ambiguous Interaction Restraints for docking BRU Tat^{46}SYGR(AcK)KRRQRC56 onto PCAF BRD.

Number	Ambiguous Interaction Restraints
**Dock1**	BRU Tat AcK50 → PCAF BRD *F748*
**Dock 2**	BRU Tat AcK50 → PCAF BRD *F748*, *V752*, *Y809*
**Dock 3**	BRU Tat AcK50 → PCAF BRD *F748*, *V752*, *Y809*, *I764*, *Y760*, *Y802*
**Dock 4**	BRU Tat AcK50 → PCAF BRD *F748*<br>BRU Tat R53 → PCAF BRD *E756*
**Dock 5**	BRU Tat AcK50 → PCAF BRD *F748*<br>BRU Tat R53 → PCAF BRD *E756*<br>BRU Tat Y47 → PCAF BRD *V763*
**Dock 6**	BRU Tat AcK50 → PCAF BRD *F748*, *V752*, *Y809*, *I764*, *Y760*, *Y802*<br>BRU Tat R53 → PCAF BRD *E756*
**Dock 7**	BRU Tat AcK50 → PCAF BRD *F748*, *V752*, *Y809*, *I764*, *Y760*, *Y802*<br>BRU Tat Y47 → PCAF BRD *V763*<br>BRU Tat R53 → PCAF BRD *E756*
**Dock 8**	BRU Tat AcK50 → PCAF BRD *F748*, *V752*, *Y809*, *I764*, *Y760*, *Y802*<br>BRU Tat Y47 → PCAF BRD *V763*<br>BRU Tat R53 → PCAF BRD *E756*<br>BRU Tat Q54 → PCAF BRD *E756*

The conformer with the lowest RMSD of residues in the interaction interface (iRMSD) respecting the NMR structure was chosen (Figure S5).

Figure S5. Cartoon representatives in seven clusters of AcK50-BRU $Tat_{full\text{-}length}$, residues 46–56 are shown in red and AcK50 in sticks.

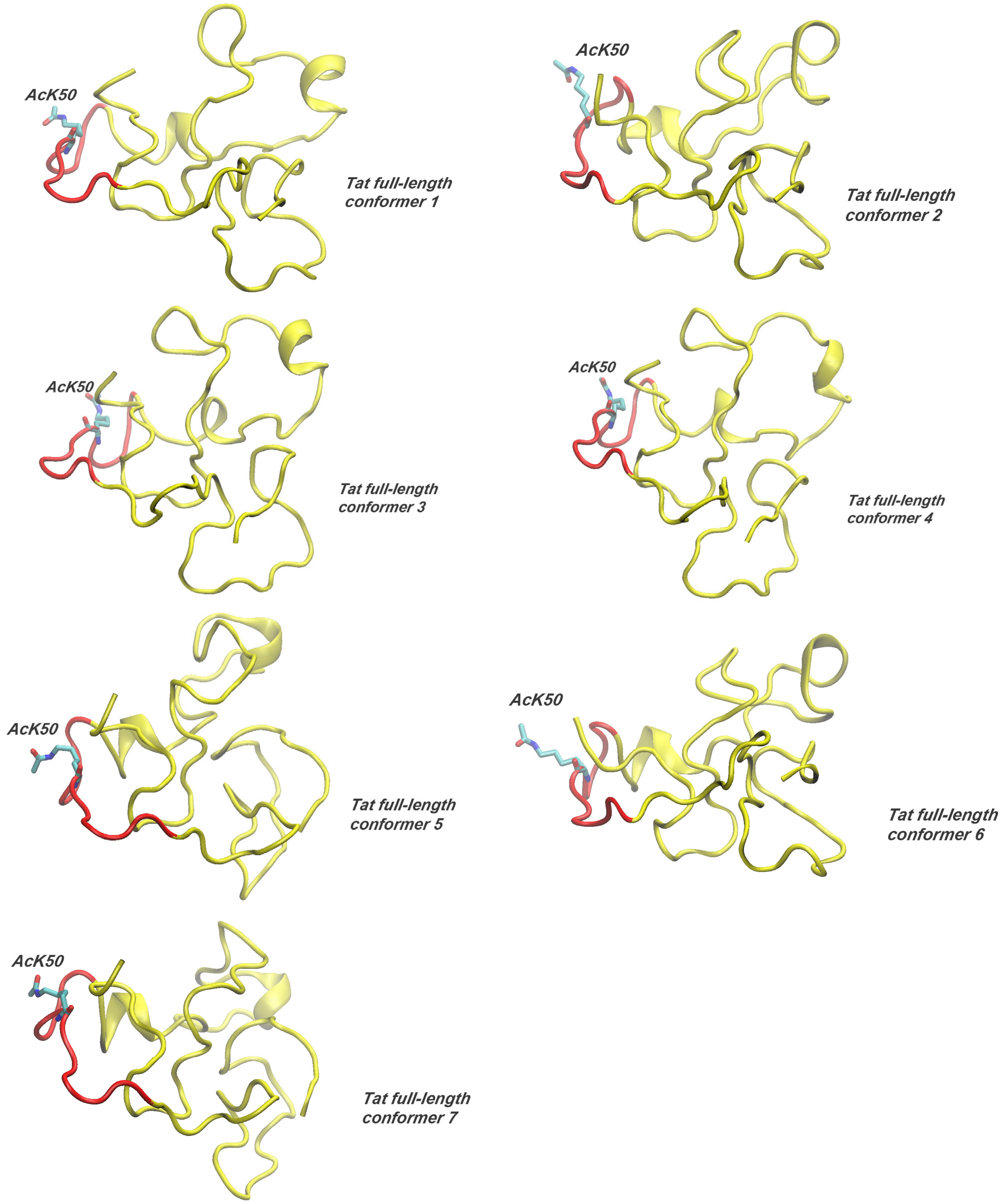

Our model reproduced almost all of the HCs present in the NMR structure [26]. The only hydrophobic contact not reproduced involved AcK50 in BRU Tat and *I764* in PCAF BRD. However, in the NMR structure, PCAF BRD *I764* is a high mobility residue [26], suggesting that this interaction might be not as strong as other hydrophobic interactions at the protein/protein interface.

Figure S6. The structure of our predicted Tat[46]SYGR(AcK)KRRQRC[56].PCAF complex is superimposed to that obtained by NMR [26]. AcK50 is shown in sticks. The interface between the peptide and the protein is represented as a grid. The van de Waals surface is represented in dots.

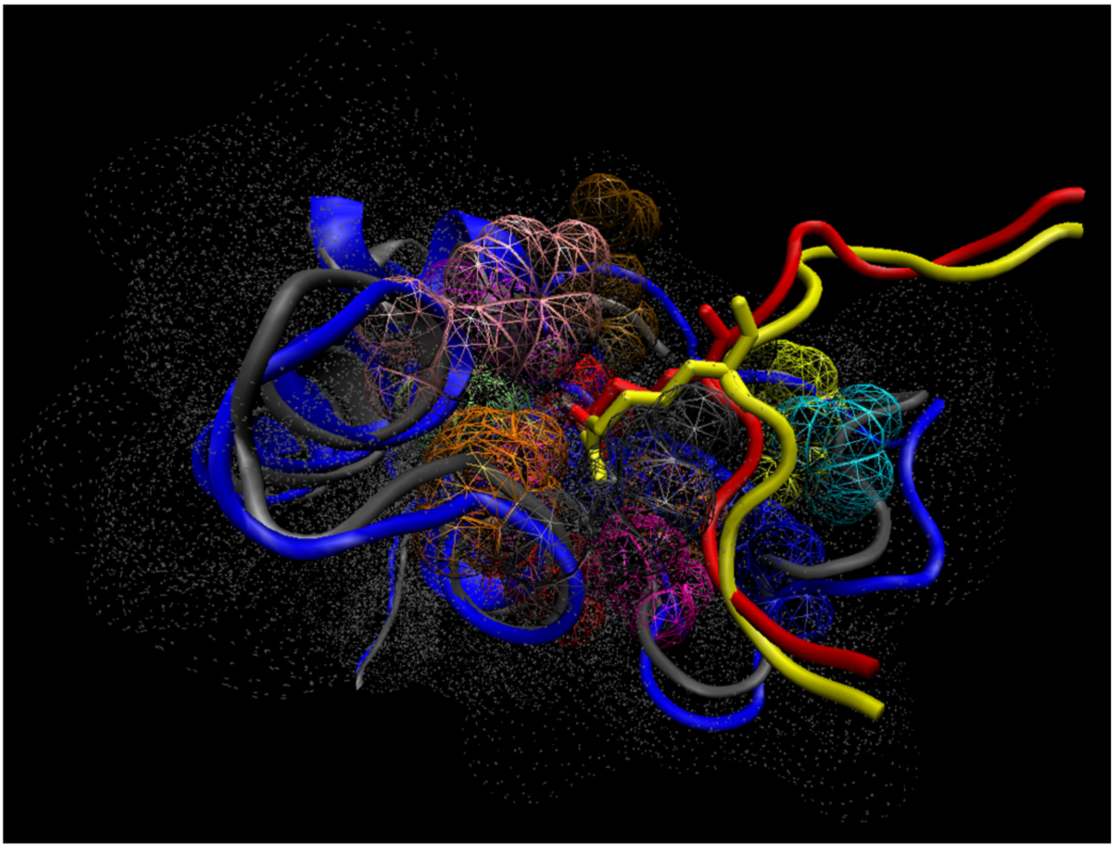

S4. MD Simulation of Tat.PCAF BRD Complex

Figure S7. MD simulation of Tat.PCAF BRD in water: RMSD of the complex plotted as a function of simulated time.

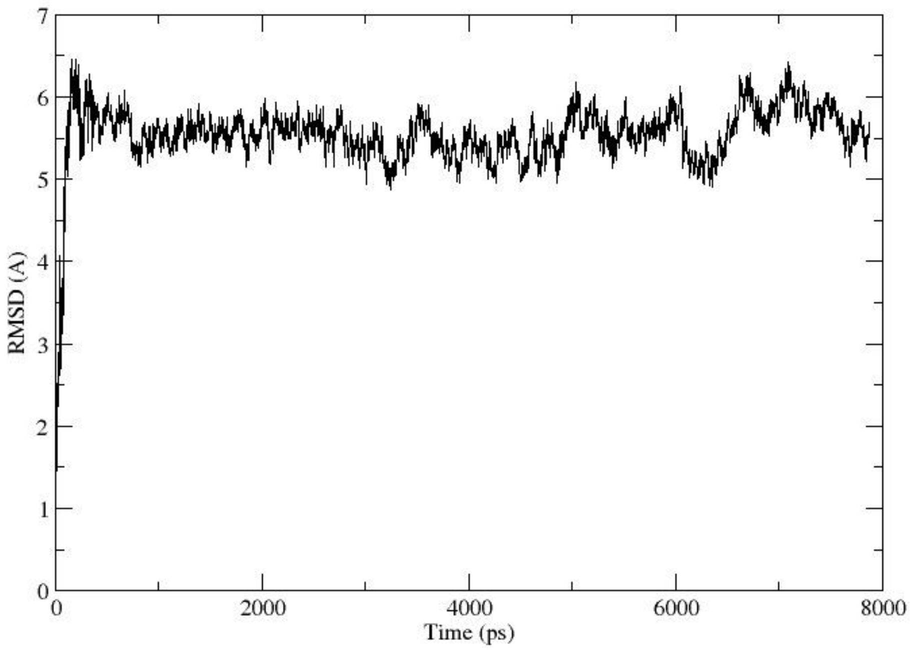

Table S3. Hydrophobic contacts observed for the representative structures of clusters 2, 3 and 4 from docked structures in comparison with *in vitro* experimental works for Tat^{46}SYGR(AcK)KRRQRC^{56} (synthesis) [15] or *in vivo* for full-length acetylated Z2 Tat with PCAF BRD [16]. (black = agreement; red = new contacts). The representative structure of cluster 1 is discussed in the main text. Coverage in the last column is the percentage of the occurrence of HCs over the representative structures.

**Effect to Tat.PCAF binding**	**Mutants**	**In contact with**	**BRU Tat.PCAF representative structures**	**Coverage**
inhibiting binding [15]	AcK50A	*F748,V752,Y760,Y802, I764, Y809*	*F748, V752, Y760, Y802*	100%
			I764, Y809, N798	75%
			K753	100%
inhibiting binding [15]	*V763*	Y47	Absent	
	Y47A	*V763*	*P804*	75%
			S807	100%
			E808	50%
			Y809	25%
	Q54A	*E756*	*E756*	50%
	E756A	Q54	R53, Q54	100%
strongly diminishing the binding [15]	R53E	*E756*	*E756, K753*	100%
			T755,	75%
			R754	25%
	F748A	Tat^{46}SYGR(AcK)KRRQRC^{56}	AcK50	100%
	V752A	Tat^{46}SYGR(AcK)KRRQRC^{56}	AcK50, R51	100%
	Y802A	Tat^{46}SYGR(AcK)KRRQRC^{56}	AcK50	100%
	Y809A	Tat^{46}SYGR(AcK)KRRQRC^{56}	AcK50, S46	75%

Table S3. *Cont.*

**Effect to Tat.PCAF binding**	**Mutants**	**In contact with**	**BRU Tat.PCAF representative structures**	**Coverage**
diminishing the binding [15]	R49A	PCAF BRD	*P747*	50%
			E750	80%
			Y809	25%
	K51A	PCAF BRD	*E750, V752*	100%
			K753	75%
			P751	50%
			P747, Y809, T75	25%
	R52A	PCAF BRD	*K753, E756*	50%
diminishing the binding [16]	*Y760D*	AcK50	AcK50	100%
	Y761D	AcK50	AcK50	25%
no effect [15]	*W746A*		Absent	
	D769A		K51	25%
	C799A		Absent	
	N803A		Absent	
	E750A		R49	100%
			K51	75%
	T755A		R53	100%
			K51	25%
	I764A		AcK50	75%
	N798A		AcK50	50%

Table S4. Selected intermolecular HBs in the representative BRU Tat.PCAF models by docking. The residues with asterisk form new HBs. Coverage in the last column is the percentage of the occurrence of HCs over the representative structures.

**Donor**	**Acceptors**	**Avg. Dist (Å)**	**Coverage**
AcK50 NZ	*Y809 OH*	3.3	25%
AcK50 NZ	*Y802 OH*	2.8	25%
Y760 OH	AcK50 OZ	2.7	25%
Q54 N	*E756 OE1*	2.8	25%
Q54 N	*E756 OE2*	2.8	25%
R53 NH1	*E756 OE1*	2.7	75%
K753 N	AcK50 O	3.2	25%
R49 NH2*	*E750 OE1**	3.3	25%
R49 NH1*	*E750 OE1**	2.7	50%
K51 NZ	*E750 OE2*	2.6	25%
K51 NZ	*P747 O*	2.8	25%
K51 NZ	*D769 OD1*	3.0	25%

S5. Tat in Complex with Cellular Partners: The P-TEFb Case

Structural information on full length HIV-1 Tat in complex with cellular partners is not available. Complexes exist instead for Tat peptide [15,17–19] with the length ranges from 9 amino acids [17] to 49 amino acids [19] (Figure S9). HIV-1 Tat peptide is always in an unfolded state [15,17,18], with exception of the complex with P-TEFb, where HIV-1 Tat adopts an α-helix [19]. The same docking protocol discussed in the main text was successfully applied also for Tat.P-TEFb complex (Figure S8 and Table S5). The best structure from the most populated cluster after docking reproduced well most of the HCs and HBs comparing to X-ray structure (PDBID 3MI9 [19]), with backbone RMSD of 1.8 Å.

Figure S8. Molecular representation of Tat.P-TEFb model and interactions. HCs are shown in the dot lines. Residues are in sticks with oxygen and nitrogen atoms in red and blue, respectively. (**a**) Superposition of Tat in complex with P-TEFb X-ray structure, PDBID 3MI9 [19] (red, orange, grey for Tat, CDK9 and CycT1, respectively) with Tat.PTEFb model (magenta, green, cyan for Tat, CDK9 and CycT1, respectively). The backbone RMSD for the two complexes is 1.8 Å. (**b**) Intermolecular HBs between Tat (magenta) and CDK9 (green) in Tat.P-TEFb model. (**c**) Selected intermolecular HBs between Tat (amgenta) and CycT1 (cyan) in Tat.P-TEFb model.

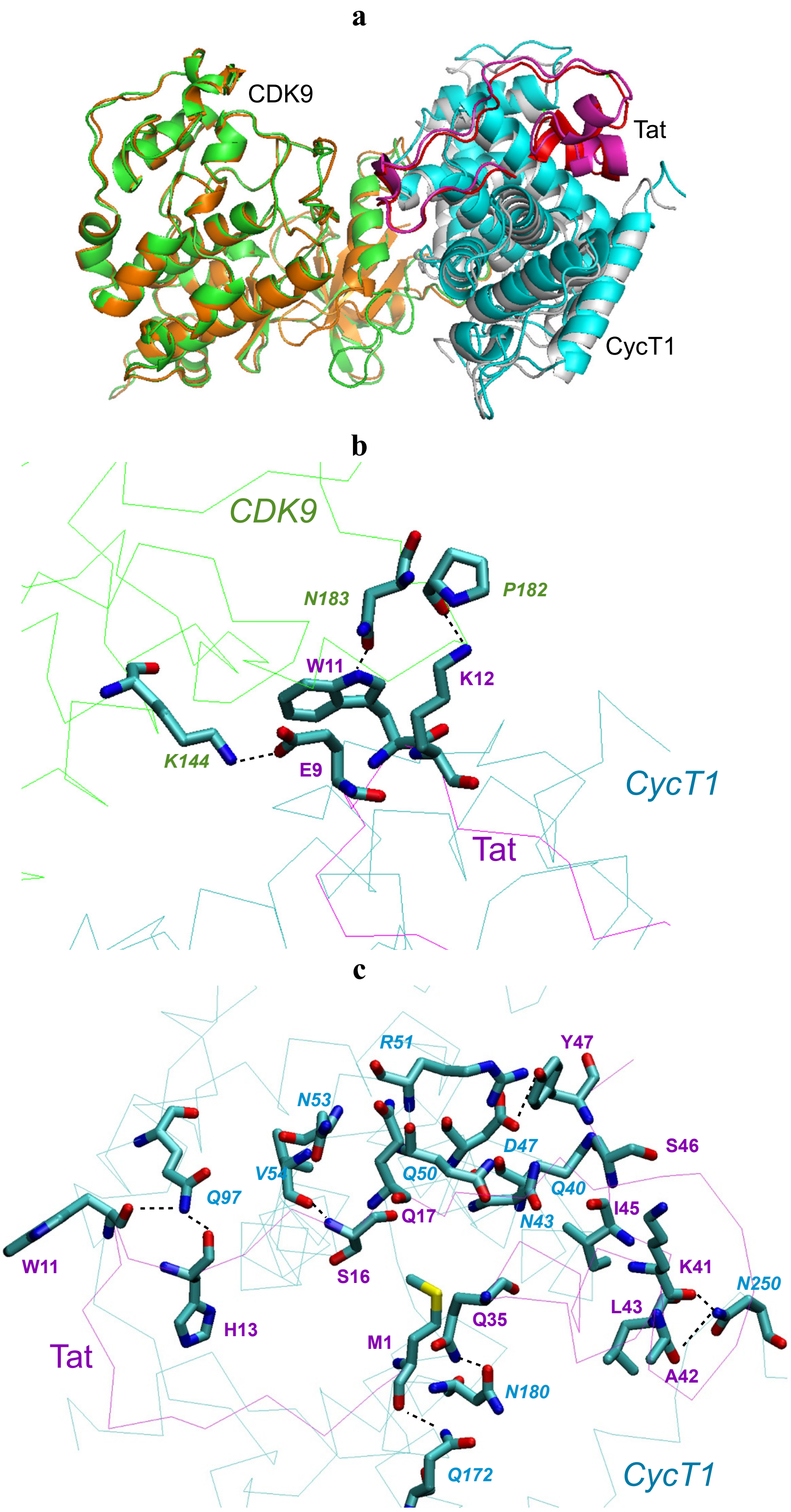

Table S5. Intermolecular HBs and hydrophobic contacts between Tat.P-TEFb model comparing to Tat and P-TEFb in X-ray structure. The residues with star are not consistent with HBs found in X-ray structure [19].

**Tat**	**CycT1**	**Distance (Å) in X-ray structure**	**Distance (Å) in Tat.P-TEFb model**
M1	Q172	3.0	3.0
W11	Q97	3.6	3.9 (*)
H13	Q97	2.8	2.8
S16	V54	2.9	2.7
S16	V54	2.9	3.4
Q17	Q50	3.4	2.8
Q17	R51	3.5	2.4
Q17	N53	3.0	3.6
Q35	N180	2.9	2.9
K41	N250	2.5	2.7
A42	N250	3.7	3.4
L43	N250	3.2	3.8 (*)
I45	N43	3.6	3.6
S46	Q40	3.2	2.8
Y47	D47	2.3	2.7
**Tat**	**CDK9**	**Distance (Å) in X-ray structure**	**Distance (Å) in Tat.P-TEFb model**
G9	K144	3.3	2.9
W11	N183	3.1	2.7
K12	P182	3.3	2.8

Figure S9. Molecular representation of HIV-1 Tat in complex with cellular partners [15,17–19]. The PDBIDs are shown in the bar with the starting and ending position of Tat (red) in the different complexes.

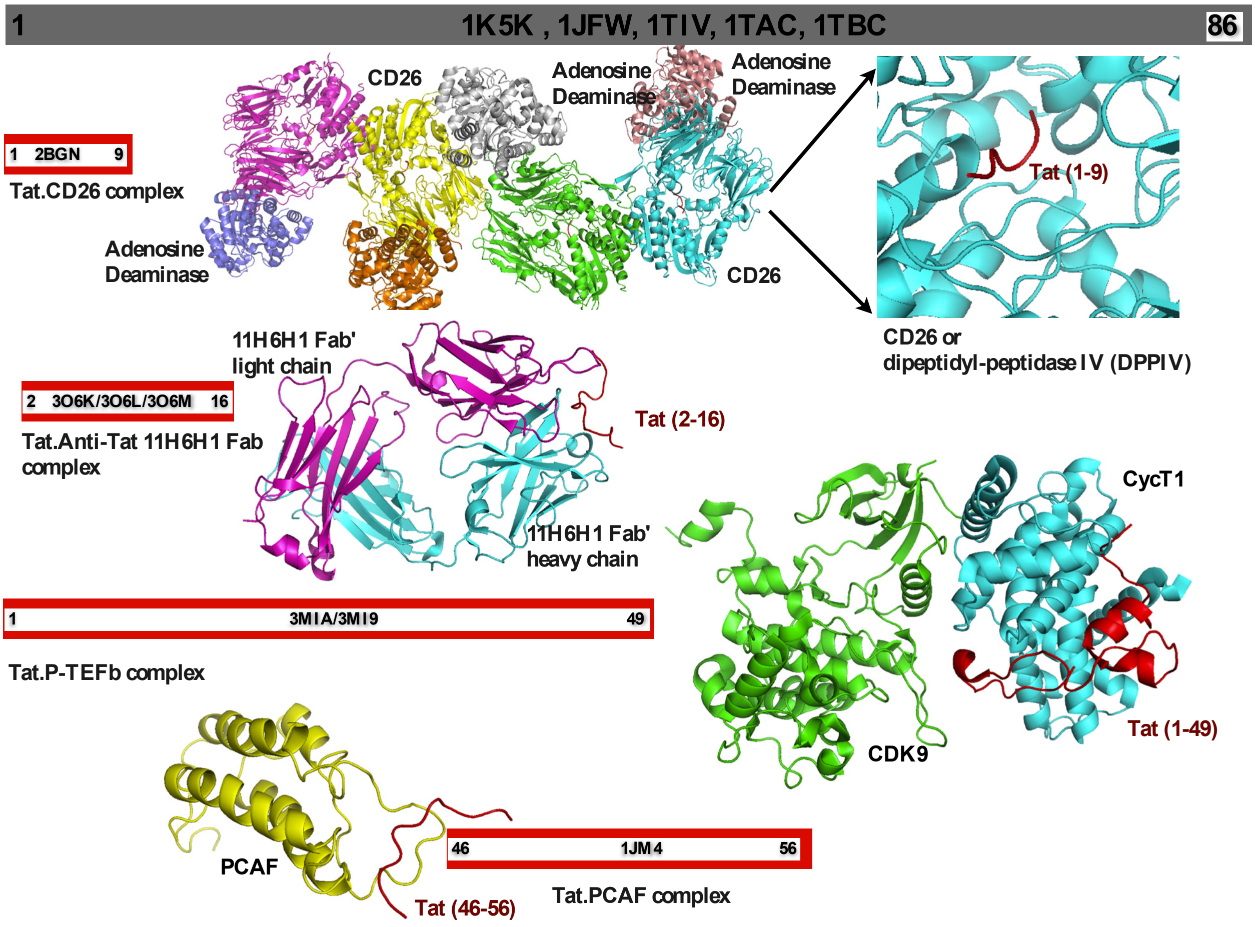

References

1. Thompson, J.D.; Higgins, D.G.; Gibson, T.J. CLUSTAL W: improving the sensitivity of progressive multiple sequence alignment through sequence weighting, position-specific gap penalties and weight matrix choice. *Nucleic Acids Res.* **1994**, *22*, 4673–4680.
2. Haynes, S.R.; Dollard, C.; Winston, F.; Beck, S.; Trowsdale, J.; Dawid, I.B. The bromodomain: a conserved sequence found in human, Drosophila and yeast proteins. *Nucleic Acids Res.* **1992**, *20*, 2603.
3. Tamkun, J.W. The role of brahma and related proteins in transcription and development. *Curr. Opin. Genet. Dev.* **1995**, *5*, 473–477.
4. Tamkun, J.W.; Deuring, R.; Scott, M.P.; Kissinger, M.; Pattatucci, A.M.; Kaufman, T.C.; Kennison, J.A. brahma: A regulator of Drosophila homeotic genes structurally related to the yeast transcriptional activator SNF2/SWI2. *Cell* **1992**, *68*, 561–572.
5. Waterhouse, A.M.; Procter, J.B.; Martin, D.M.; Clamp, M.; Barton, G.J. Jalview Version 2—A multiple sequence alignment editor and analysis workbench. *Bioinformatics* **2009**, *25*, 1189–1191.
6. Jorgensen, W.L.; Chandrasekhar, J.; Madura, J.D.; Impey, R.W.; Klein, M.L. Comparison of Simple Potential Functions for Simulating Liquid Water. *J. Chem. Phys.* **1983**, *79*, 926–935.

7. Makov, G.; Payne, M.C. Periodic boundary conditions in ab initio calculations. *Phys. Rev. B* **1995**, *51*, 4014–4022.
8. Darden, T.; York, D.; Pedersen, L. Particle mesh Ewald: An N [center-dot] log(N) method for Ewald sums in large systems. *J. Chem. Phys.* **1993**, *98*, 10089–10092.
9. Ryckaert, J.-P.; Ciccotti, G.; Berendsen, H.J.C. Numerical integration of the cartesian equations of motion of a system with constraints: molecular dynamics of n-alkanes. *J. Comput. Phys.* **1977**, *23*, 327–341.
10. Berendsen, H.J.C.; Postma, J.P.M.; van Gunsteren, W.F.; DiNola, A.; Haak, J.R. Molecular dynamics with coupling to an external bath. *J. Chem. Phys.* **1984**, *81*, 3684–3690.
11. Hornak, V.; Abel, R.; Okur, A.; Strockbine, B.; Roitberg, A.; Simmerling, C. Comparison of multiple Amber force fields and development of improved protein backbone parameters. *Proteins* **2006**, *65*, 712–725.
12. Machado, M.R.; Dans, P.D.; Pantano, S. Isoform-specific determinants in the HP1 binding to histone 3: insights from molecular simulations. *Amino Acids* **2010**, *38*, 1571–1581.
13. Phillips, J.C.; Braun, R.; Wang, W.; Gumbart, J.; Tajkhorshid, E.; Villa, E.; Chipot, C.; Skeel, R.D.; Kale, L.; Schulten, K. Scalable molecular dynamics with NAMD. *J. Comput. Chem.* **2005**, *26*, 1781–1802.
14. De Mori, G.M.; Colombo, G.; Micheletti, C. Study of the Villin headpiece folding dynamics by combining coarse-grained Monte Carlo evolution and all-atom molecular dynamics. *Proteins* **2005**, *58*, 459–471.
15. Mujtaba, S.; He, Y.; Zeng, L.; Farooq, A.; Carlson, J.E.; Ott, M.; Verdin, E.; Zhou, M.M. Structural basis of lysine-acetylated HIV-1 Tat recognition by PCAF bromodomain. *Mol. Cell* **2002**, *9*, 575–586.
16. Pantano, S.; Marcello, A.; Ferrari, A.; Gaudiosi, D.; Sabo, A.; Pellegrini, V.; Beltram, F.; Giacca, M.; Carloni, P. Insights on HIV-1 Tat:P/CAF bromodomain molecular recognition from in vivo experiments and molecular dynamics simulations. *Proteins* **2006**, *62*, 1062–1073.
17. Weihofen, W.A.; Liu, J.; Reutter, W.; Saenger, W.; Fan, H. Crystal structures of HIV-1 Tat-derived nonapeptides Tat-(1–9) and Trp2-Tat-(1–9) bound to the active site of dipeptidyl-peptidase IV (CD26). *J. Biol. Chem.* **2005**, *280*, 14911–14917.
18. Serriere, J.; Dugua, J.M.; Bossus, M.; Verrier, B.; Haser, R.; Gouet, P.; Guillon, C. Fab'-induced folding of antigenic N-terminal peptides from intrinsically disordered HIV-1 Tat revealed by X-ray crystallography. *J. Mol. Biol.* **2011**, *405*, 33–42.
19. Tahirov, T.H.; Babayeva, N.D.; Varzavand, K.; Cooper, J.J.; Sedore, S.C.; Price, D.H. Crystal structure of HIV-1 Tat complexed with human P-TEFb. *Nature* **2010**, *465*, 747–751.
